# 诱导治疗失败和复发急性髓系白血病患者获得血液学缓解和生存的影响因素分析

**DOI:** 10.3760/cma.j.issn.0253-2727.2022.08.005

**Published:** 2022-08

**Authors:** 艳茹 马, 婷 赵, 玲 马, 利娟 胡, 文冰 段, 浩 江, 晓军 黄, 倩 江

**Affiliations:** 北京大学人民医院、北京大学血液病研究所、国家血液系统疾病临床医学研究中心，北京 100044 Peking University People's Hospital, Peking University Institute of Hematology, National Clinical Research Center for Hematologic Disease, Beijing 100044, China

**Keywords:** 白血病，髓系，急性, 诱导失败, 复发, 治疗结局, Leukemia, myeloid, acute, Induction failure, Relapse, Treatment autcome

## Abstract

**目的:**

探索诱导化疗失败和复发急性髓系白血病（AML）患者缓解、复发、生存的影响因素。

**方法:**

回顾性收集2008年1月1日至2021年5月1日北京大学人民医院收治的诱导化疗失败和复发AML连续病例，采用二元Logistics和Cox比例风险回归模型分析患者治疗反应和结局的影响因素。

**结果:**

180例诱导化疗失败和193例复发AML患者，最终完全缓解（CR）/CR伴血细胞未完全恢复（CRi）率分别为50.6％和40.3％；获得CR/CRi人群中，3年无复发生存（RFS）率分别为34.4％和30.4％，3年总生存（OS）率分别为40.1％和31.6％。多因素分析显示，诱导化疗失败患者中，CLAG或FLAG方案作为再诱导化疗方案、年龄<39岁和SWOG预后分层低危和缓解率高相关；复发患者中，男性、继发AML、SWOG预后分层高危、12个月内复发和复发时骨髓原始细胞比例≥20％和缓解率低相关（*P*值均<0.05）。对于获得CR/CRi的患者，移植是影响RFS和OS的共同因素；此外，SWOG预后分层与诱导化疗失败患者的OS率显著相关，获得CR而非CRi与复发患者的RFS率相关，男性复发患者的OS率低于女性。

**结论:**

对于诱导化疗失败和复发AML患者，再诱导化疗方案、SWOG预后分层、年龄、性别、继发或初发AML、首次缓解距复发时间、复发时骨髓中原始细胞比例是影响缓解的独立因素；移植、SWOG预后分层、缓解时血细胞恢复状态和性别影响获得缓解者的生存。

## Abstract (en)

**Objective:**

This study aimed to explore variables associated with remission rate and survival in patients with acute myeloid leukemia（AML）after induction failure and relapse.

**Methods:**

Data of 373 consecutive patients with AML were analyzed after induction failure and relapse. Binary logistics and the Cox model regression were used to identify variables associated with remission rate and outcomes.

**Results:**

In patients with AML after induction failure and relapse, the total CR+CRi rates were 50.6％ and 40.3％, respectively; among those who achieved CR/CRi, the 3-year RFS rates were 34.4％ and 30.4％, respectively, and the 3-year overall survival rates were 40.1％ and 31.6％, respectively. In the multivariate analyses, using CLAG or FLAG regimen as a re-induction chemotherapy regimen, age <39 years and SWOG low-risk were significantly associated with higher remission rates in patients with induction failure. Male, secondary AML, SWOG high-risk, the interval from the first remission to relapse within 12 months, and bone marrow blasts ≥20％ at the time of relapse were significantly associated with lower remission rates in relapsed patients. Transplantation was significantly associated with prolonged relapse-free survival and overall survival in patients achieving hematologic remission; the SWOG low-risk group was significantly associated with longer overall survival in those with induction failure; and achieving CR（not CRi）or having female gender was associated with longer RFS or overall survival in relapsed patients.

**Conclusion:**

Reinduction chemotherapy regimen, age, gender, SWOG risk, secondary AML, the interval from the first remission to relapse, and bone marrow blast percentage at the time of relapse were significantly associated with remission rates in the patients with AML after induction failure and relapse. Transplantation, SWOG low-risk, achieving CR, or female gender were associated with longer survivals in those achieving remission.

难治复发急性髓系白血病（AML）缓解率低、预后不良，是白血病治疗的重要挑战。目前无标准挽救性治疗方案，公认的首选治疗为参加临床试验或靶向药物为主的治疗；若无以上可及方案，可耐受强化疗者建议接受含有高剂量阿糖胞苷（HDAC）的化疗[1]–[3]。获得形态学缓解后，尽快行异基因造血干细胞移植（allo-HSCT）。此外，新药、新的治疗策略的问世也给难治复发AML患者带来更多选择和希望。虽然国外已有多项研究发现首次缓解持续时间[4]–[6]、年龄[5]、染色体核型[5]–[7]、FLT-ITD突变[6],[8]、是否接受移植[5]等因素影响难治复发AML患者的缓解率和总体结局，但国内多为分析单一药物、有限种类多药组合方案或移植用于难治复发AML挽救治疗的报道。因此，我们回顾性分析本中心收治的373例诱导化疗失败或复发AML（非急性早幼粒细胞白血病）患者连续病例资料，探讨挽救性诱导化疗的缓解率和总体结局，并分析相关影响因素。

## 病例与方法

一、病例

回顾性收集2008年1月1日至2021年5月1日于北京大学人民医院接受挽救性化疗的373例诱导化疗失败或复发AML患者。纳入标准：①年龄18～65岁；②经骨髓细胞形态学、免疫学、细胞遗传学、分子生物学明确诊断AML；③至少接受过1个疗程标准诱导化疗（IA10或HAA方案）未达部分缓解（PR）、2个疗程化疗（至少1个疗程标准诱导化疗或含有HDAC的方案）未达完全缓解（CR）或CR伴血细胞未完全恢复（CRi），或缓解后再次出现血液学复发[3]，[9]。标准诱导化疗方案：根据中国AML诊疗指南[10]，所有患者入院后均接受标准诱导化疗，标准剂量方案包括：IA10［去甲氧柔红霉素（IDA）10 mg/m^2^，第1～3天；阿糖胞苷（Ara-C）100 mg/m^2^，第1～7天］、HAA［高三尖杉酯碱（HHT）2 mg/m^2^，第1～7天；阿克拉霉素（Acla）20 mg，第1～7天；Ara-C 100 mg/m^2^，第1～7天］[10]。排除标准：①急性早幼粒细胞白血病；②接受减低剂量化疗或姑息治疗；③参加临床试验患者。

二、实验室检查

初诊时进行骨髓细胞形态学、免疫分型、分子生物学检测、细胞遗传学分析。骨髓细胞免疫表型分析方法见文献[11]–[12]。细胞遗传学分析：采用G显带法分析骨髓染色体核型，根据《人类细胞遗传学国际命名体制（ISCN,1995）》进行核型描述；参考美国西南肿瘤协作组（SWOG）标准[13]进行危险度预后分层。分子生物学检测：采用实时荧光定量PCR方法检测RUNX1-RUNX1T1、CBFβ-MYH11、MLL相关融合基因、BCR-ABL转录本水平，以及NPM1基因突变、FLT3-ITD基因突变[14]。

三、治疗

1. 挽救性治疗：根据《复发难治性急性髓系白血病中国诊疗指南（2017年版）》[15]，首选临床试验，无法进入临床试验的患者，接受挽救性化疗，挽救性化疗方案包括：CLAG方案（克拉屈滨5 mg/m^2^，第1～5天；Ara-C 1 g/m^2^，第1～5天；G-CSF 300 µg/m^2^，第0～5天）、FLAG方案［氟达拉滨（Flu）25～30 mg/m^2^，第1～5天；Ara-C 1 g/m^2^，第1～5天；G-CSF 300 µg/m^2^，第0～5天］、CAG方案（G-CSF 150 µg/m^2^，每12 h 1次，第0～14天；Acla 20 mg/d，第1～4天；Ara-C 20 mg/m^2^，分2次皮下注射，第1～14天）、HDAC为主的方案、IA10方案、HAA方案及其他方案，如MEA（米托蒽醌+依托泊苷+Ara-C）、MA（米托蒽醌+Ara-C）、EA（依托泊苷+Ara-C）方案等。分别以IA和HAA方案作为初始诱导治疗方案的患者，可以选择原诱导方案以外的上述诱导方案。

2. 缓解后的治疗：获得CR/CRi后，以原方案或HDAC为主的方案巩固治疗。根据有无供者、社会经济、自身状况、个人意愿等因素，选择尽快行allo-HSCT（方案参见文献[16]–[17]）或继续巩固化疗。

四、评估内容和标准

根据2017年欧洲白血病网（ELN）指南[18]，具体定义如下：①CR：骨髓原始细胞<5％，外周血无原始细胞，无含Auer小体的原始细胞，无髓外白血病，ANC≥1.0×10^9^/L且PLT≥100×10^9^/L；②CR伴CRi：CR伴ANC或PLT未恢复（ANC<1.0×10^9^/L或PLT<100×10^9^/L）；③PR：血细胞恢复达CR，骨髓原始细胞比例5％～25％且较治疗前下降至少50％。

主要研究终点为缓解率，包括CR和CRi。次要研究终点为生存结局，包括总生存（OS）和无复发生存（RFS）。OS期定义为开始挽救性治疗至死亡或随访截止的时间。RFS期定义为获得CR/CRi患者，从CR/CRi至复发或任何原因死亡或随访截止的时间。早期死亡定义为在可评估疗效前死亡。

五、随访

主要采用门诊随访和电话随访方式，随访截止日期为2021年6月1日。

六、统计学处理

采用描述性统计，分析患者基本特征。缓解率的比较应用卡方检验及Fisher确切概率法。针对挽救化疗的缓解率，使用二元Logistic模型进行单因素分析，所有单因素分析中*P*<0.2的因素纳入Logistic回归模型进行多因素分析。影响RFS和OS的因素采用Kaplan-Meier生存曲线进行Log-rank检验，所有单因素分析中*P*<0.2的因素代入Cox回归模型（LR向后法）进行多因素分析。*P*<0.05为差异有统计学意义。数据分析应用SPSS 21.0软件。

## 结果

一、患者特征

本研究包括373例接受挽救治疗的AML连续病例，180例为诱导化疗失败，193例为复发。180例诱导化疗失败患者诊断AML后首次诱导方案为IA10方案154例（85.6％），HAA方案26例（14.4％），其中首疗程标准诱导化疗（IA10或HAA）未达PR者163例，2个疗程未达CR或CRi（CR/CRi）者17例。193例复发患者中，中位复发时间为缓解后8（2～76）个月，1年内复发者128例（66.3％），≥1年复发者65例（33.7％）。

373例诱导化疗失败或复发患者中，333例为初发AML，40例（10.7％）为继发白血病。男212例（56.8％），中位年龄43（18～65）岁。按SWOG危险分层低危组、中危组、高危组和未确定组分别为47例（12.6％）、185例（49.6％）、89例（23.9％）和52例（13.9％）。180例诱导化疗失败和193例复发患者中，RUNX1-RUNX1T1融合基因阳性分别为14例（7.8％）和18例（9.3％），CBFβ-MYH11阳性分别为3例（1.7％）和15例（7.8％），MLL基因重排分别为18例（10％）和9例（4.7％），FLT3-ITD突变阳性（包括NPM1阳性或阴性）分别为56例（31.1％）和29例（15.0％），NPM1阳性伴FLT3-ITD阴性分别为22例（12.2％）和53例（27.5％）。详见表1。

**表1:** 诱导失败及复发急性髓系白血病患者的一般特征

一般特征	诱导化疗失败（180例）	复发（193例）
年龄［岁，*M*（范围）］	39（18~65）	45（18~65）
男性［例（％）］	112（62.2）	100（51.8）
诊断时		
WBC［×10^9^/L，*M*（范围）］	17（1~379）	21（1~142）
HGB［g/L，*M*（范围）］	78（39~145）	85（43~158）
PLT［×10^9^/L，*M*（范围）］	48（5~671）	39（2~444）
骨髓原始细胞［％，*M*（范围）］	66（4~96）	62（7~98）
外周原始细胞［％，*M*（范围）］	60（0~98）	54（0~98）
诱导失败或复发时		
WBC［×10^9^/L，*M*（范围）］	2（0~206）	3（1~78）
HGB［g/L，*M*（范围）］	79（50~136）	103（60~182）
PLT［×10^9^/L，*M*（范围）］	52（3~506）	90（3~529）
骨髓原始细胞［％，*M*（范围）］	38（6~97）	21（5~97）
外周原始细胞［％，*M*（范围）］	10（0~96）	2（0~98）
疾病状态［例（％）］		
初发	159（88.3）	174（90.2）
继发	21（11.7）	19（9.8）
SWOG预后分层［例（％）］		
低危	16（8.9）	31（16.1）
中危	78（43.3）	107（55.4）
高危	59（32.8）	30（15.5）
未知	27（15.0）	25（13.0）
FAB分型［例（％）］		
M_0_	6（3.3）	2（1.0）
M_1_	3（1.7）	2（1.0）
M_2_	104（57.8）	114（59.1）
M_4_	39（21.7）	38（19.7）
M_5_	22（12.2）	33（17.1）
M_6_	3（1.7）	2（1.0）
不明确	3（1.7）	2（1.0）

注：SWOG：美国西南肿瘤协作组

二、诱导化疗失败患者接受挽救化疗的疗效及其影响因素

1. 再诱导化疗方案和缓解率：180例患者中，首疗程挽救化疗方案分别为CLAG 14例（7.8％）、FLAG 23例（12.8％）、CAG 85例（47.2％）、HAA 20例（11.1％）、HDAC 9例（5.0％）、其他29例（16.1％）。

随访期内，2例（1.1％）早期死亡（FLAG和CAG方案组各1例），在可评估的178例患者中，接受中位2（1～5）个疗程再诱导化疗，45例（25.3％）在首疗程挽救化疗中获得CR/CRi，其中40例获得CR（22.5％）。首疗程挽救化疗方案获得CR+CRi率由高到低依次为CLAG（71.4％）、FLAG（45.5％）、CAG（22.6％）、其他（13.8％）、HDAC（11.1％）、HAA（5.0％）。最终，90例（50.6％）获得CR/CRi，其中78例（43.8％）获得CR。178例患者共接受296次挽救性化疗，所有挽救化疗方案获得CR+CRi率由高到低依次为CLAG（55.9％）、FLAG（42.6％）、CAG（29.9％）、HDAC（26.1％）、HAA（14.7％）、其他（13.0％）。

为探讨患者特征与获得缓解的相关性，分析首疗程挽救化疗缓解率纳入的变量包括：初诊时年龄、性别、SWOG分层、AML类型（初发/继发）、诱导失败时骨髓和外周血原始细胞比例、NPM1突变、FLT3-ITD突变、首疗程挽救化疗方案；分析最终缓解率时纳入除挽救化疗方案以外的上述变量。

多因素分析显示，首疗程挽救化疗方案与首疗程挽救化疗后获得CR+CRi（*P*＝0.001）和获得CR（*P*＝0.001）显著相关，GLAG和FLAG方案显著优于其他方案。年龄≥39岁患者总CR率低（*P*＝0.040），SWOG预后分层低危患者总CR率高（表2）。

**表2:** 影响诱导化疗失败及复发急性髓系白血病（AML）患者缓解率的多因素分析

因素	首疗程CR+CRi		首疗程CR		总CR+CRi		总CR	
	*OR*（95%*CI*）	*P*值	*OR*（95%*CI*）	*P*值	*OR*（95%*CI*）	*P*值	*OR*（95%*CI*）	*P*值
诱导失败AML								
年龄								0.040
<39岁（参照）							1	
≥39岁							0.5（0.3~1.0）	
疾病状态						0.099		0.098
初发（参照）					1		1	
继发					0.4 (0.2~1.2）		0.4（0.1~1.2）	
SWOG预后分层								0.109
低危（参照）							1	
中危							0.3（0.1~0.9）	0.034
高危							0.2（0.1~0.7）	0.010
未知							0.2（0.1~1.0）	0.048
化疗方案		0.001		0.001				
CLAG（参照）	1		1					
FLAG	0.3（0.1~1.4）	0.132	0.6（0.2~2.4）	0.495				
CAG	0.1（0.0~0.4）	0.001	0.2（0.1~0.7）	0.008				
HAA	0.0（0.0~0.2）	0.001	0.0（0.0~0.4）	0.005				
HDAC	0.1（0.0~0.5）	0.014	0.1（0.0~1.0）	0.047				
其他	0.1（0.0~0.3）	0.001	0.1（0.0~0.3）	0.001				
复发AML								
性别		0.028		0.035				
女（参照）	1		1					
男	0.5（0.2~0.9）		0.5（0.2~0.9）					
疾病状态		0.018		0.027		0.078		0.094
初发（参照）	1		1		1		1	
继发	0.1（0.0~0.7）		0.1（0.0~0.8）		0.3（0.1~1.1）		0.3（0.1~1.2）	
SWOG危险分层		0.100		0.063		0.041		0.016
低危（参照）	1		1		1		1	
中危	1.0（0.4~2.5）	0.935	0.8（0.3~2.1）	0.715	0.6（0.3~1.5）	0.290	0.5（0.2~1.2）	0.103
高危	0.2（0.1~0.9）	0.034	0.1（0.0~0.6）	0.010	0.1（0.0~0.5）	0.005	0.1（0.0~0.4）	0.001
未知	0.8（0.3~2.9）	0.793	0.8（0.2~2.6）	0.651	0.6（0.2~2.0）	0.443	0.5（0.1~1.5）	0.207
缓解至复发时间		0.003		0.047		<0.001		0.016
≥12个月（参照）	1		1		1		1	
<12个月	0.4（0.2~0.7）		0.5（0.2~1.0）		0.3（0.2~0.6）		0.4（0.2~0.8）	
复发时骨髓原始细胞		0.001		0.001		0.003		0.002
<20%（参照）	1		1		1		1	
≥20%	0.3（0.2~0.6）		0.3（0.2~0.6）		0.4（0.2~0.7）		0.3（0.2~0.7）	

注：CR：完全缓解；CRi：CR伴血细胞未完全恢复；SWOG：美国西南肿瘤协作组；CLAG：克拉屈滨+阿糖胞苷（Ara-C）+G-CSF；FLAG：氟达拉滨+Ara-C+G-CSF；CAG：G-CSF+阿克拉霉素+Ara-C；HAA：高三尖杉酯碱+Ara-C+阿柔比星；HDAC：大剂量阿糖胞苷

2. 生存情况：88例未获得CR/CRi患者中，21例（23.9％）接受了allo-HSCT，中位OS期为6（95％*CI* 4～8）个月。

90例获得CR/CRi的患者中，存活患者中位随访时间为缓解后20.5（1～78）个月，53例（58.9％）接受了allo-HSCT。45例（50.0％）在缓解后中位6（1～28）个月时复发，48例在缓解后中位9.5（1～66）个月时死亡，其中38例死于复发，10例死于感染或移植相关并发症（7例为移植后）。中位RFS期为15（95％*CI* 9～21）个月，3年RFS率34.4％（95％*CI* 21.9％～46.9％）；中位OS期为23（95％*CI* 14～32）个月，3年OS率40.1％（95％*CI* 28.3％～51.9％）。移植组和持续化疗组CR后3年复发率分别为56.0％（95％*CI* 38.8％～73.2％）和80.7％（95％*CI* 65.0％～96.4％）（*P*＝0.001），3年OS率分别为51.9％（95％*CI* 35.8％～68.0％）和21.9％（95％*CI* 6.8％～37.0％）（*P*＝0.002）。

为探讨患者特征与生存的相关性，RFS和OS纳入的变量包括：初诊时的年龄、性别、SWOG预后分层、AML类型（初发/继发）、诱导失败时骨髓和外周血原始细胞比例、NPM1突变、FLT3-ITD突变、移植、治疗反应（CR/CRi）。

获得缓解人群中，多因素分析结果显示，持续化疗未移植与较短的RFS期（*P*＝0.002）和OS期（*P*<0.001）相关，SWOG预后分层与OS显著相关（表3）。

**表3:** 影响获得CR/CRi患者生存的多因素分析

因素	无复发生存		总生存	
	*HR*（95％ *CI*）	*P*值	*HR*（95％ *CI*）	*P*值
诱导失败AML				
SWOG预后分层		0.099		0.016
低危（参照）	1		1	
中危	1.4（0.5~3.8）	0.454	3.9（1.1~13.5）	0.030
高危	1.9（0.7~5.2）	0.209	7.3（2.9~26.2）	0.002
未知	0.3（0.1~1.7）	0.181	4.4（1.0~20.5）	0.058
移植		0.002		<0.001
否（参照）	1		1	
是	0.4（0.2~0.7）		0.3（0.1~0.5）	
复发AML				
性别				0.057
男（参照）			1	
女			0.6（0.3~1.0）	
挽救疗效		0.055		
获CRi（参照）	1			
获CR	0.5（0.2~1.0）			
移植		0.001		0.001
否（参照）	1		1	
是	0.3（0.2~0.6）		0.3（0.2~0.6）	

注：SWOG：美国西南肿瘤协作组；CR：完全缓解；CRi：CR伴血细胞未完全恢复；AML：急性髓系白血病

三、复发患者接受挽救化疗的疗效及其影响因素

1. 再诱导方案和缓解率：193例患者中，首疗程挽救化疗方案分别为CLAG 23例（11.9％）、FLAG 13例（6.7％）、CAG 46例（23.8％）、HAA 33例（17.1％）、HDAC 9例（4.7％）、其他69例（35.8％）。缓解1年后复发者中，13例（20.0％）采用初诊AML时的首个诱导缓解方案：IA10 6例，HAA 1例，CAG 4例，MA 2例。

随访期内，2例在再诱导过程中早期死亡（HAA和CAG方案组各1例）。在可评估的191例患者中，中位接受2（1～9）个疗程再诱导化疗，68例（35.6％）在首疗程挽救化疗中获得CR/CRi，其中57例（29.8％）达CR。首疗程挽救化疗方案CR+CRi率由高到低依次为CLAG（52.2％）、HDAC（44.4％）、CAG（42.2％）、FLAG（38.5％）、HAA（31.3％）、其他（26.1％）。最终77例（40.3％）获得CR/CRi，其中64例（33.5％）获得CR。191例患者共接受304次挽救性化疗，再诱导方案获得CR+CRi率由高到低依次为CLAG（43.3％）、HDAC（41.7％）、CAG（34.8％）、FLAG（31.6％）、HAA（20.0％）、其他（15.7％）。

为探讨患者特征与获得缓解的相关性，分析首疗程挽救化疗缓解率纳入的变量包括：初诊时年龄、性别、SWOG预后分层、AML类型（初发/继发）、首次缓解距复发的时间（12个月为界）、复发时骨髓和外周血原始细胞比例、首疗程挽救化疗方案。分析最终缓解率时，纳入除挽救化疗方案以外的上述变量。

多因素分析显示，SWOG预后分层高危、缓解至复发时间<12个月、复发时骨髓原始细胞比例≥20％与首疗程诱导后和最终获得CR+CRi和CR均显著相关。此外，男性和继发AML是首疗程诱导化疗后获得CR+CRi和CR的不利因素（表2）。

2. 生存情况：114例未获缓解者中，9例（7.9％）接受了allo-HSCT，中位OS期为4（95％ *CI* 3～5）个月。

77例获得CR/CRi的患者中，存活患者中位随访时间为缓解后25（1～102）个月，34例（44.2％）接受了allo-HSCT。47例（61.0％）在中位6（1～28）个月复发，46例在缓解后中位9（2～60）个月死亡，其中43例死于复发，3例死于感染（2例为移植后）。中位RFS期为10（95％*CI* 8～12）个月，3年RFS率30.4％（95％*CI* 18.8％～42.0％）；中位OS期为22（95％*CI* 13～31）个月，3年OS率31.6％（95％*CI* 19.6％～43.6％）。

为探讨患者特征与生存的相关性，RFS和OS纳入的变量包括：初诊时的年龄、性别、SWOG预后分层、AML类型（初发/继发）、首次缓解距复发的时间、复发时骨髓和外周血原始细胞比例、移植、CR/CRi。

在获得缓解的人群中，多因素分析结果显示，持续化疗未移植与较短的RFS（*P*＝0.001）和OS期（*P*＝0.001）显著相关。此外，获得CRi而非CR与较短的RFS期显著相关（*P*＝0.055），男性与较短的OS期显著相关（*P*＝0.057）（表3）。

## 讨论

既往多数研究将难治和复发AML作为一个整体分析，本研究将诱导化疗失败和复发AML分别进行分析，发现两者的缓解和预后情况及其影响因素不同。通过回顾性分析发现，经多疗程再诱导化疗，50.6％的诱导治疗失败患者和40.3％的复发患者最终获得缓解（CR+CRi），与既往文献报道相似[19]–[20]。诱导化疗失败患者中，首次挽救性化疗方案与缓解率相关，总缓解率与年龄、SWOG危险分层、是否为继发AML相关。而复发患者中，SWOG危险分层高危、缓解至复发时间≥12个月、复发时骨髓原始细胞比例≥20％、男性和继发AML与低的首疗程和总缓解率显著相关。

多项临床研究证实更强的化疗方案有助于获得缓解[21]–[24]，国内外相关指南均推荐CLAG、FLAG等含HDAC的方案作为难治复发AML除临床试验外的再诱导化疗方案。本研究结果也显示，CLAG方案缓解率最高，其次为FLAG方案，而且挽救化疗方案是诱导治疗失败患者获得缓解的独立影响因素。

在本研究中我们发现，复发时骨髓原始细胞比例≥20％在复发AML中预示着更低的缓解率。Kern等[25]早期研究报道254例接受高剂量Ara-C+米托蒽醌挽救化疗的难治复发AML患者，发现挽救化疗前骨髓原始细胞比例>80％和更短的生存期相关，但此项研究未将核型作为影响因素纳入分析。本研究在纳入SWOG危险分层的多因素分析中发现，挽救化疗前的原始细胞比例是影响复发AML预后的独立危险因素。提示复发时低肿瘤负荷可能与更好的预后相关，应定期监测骨髓，早期识别复发并积极干预。

继发AML危险分层属于高危，预后不良。本研究结果显示，诱导化疗失败及复发AML中，继发AML亦为不良预后因素，其挽救化疗缓解率低于初发AML。这与Ferguson等[26]既往研究报道一致。来自MD Anderson的一项研究[27]显示，前驱血液病史与首疗程挽救化疗的缓解率、EFS、及OS均独立相关。在新药时代，BCL-2抑制剂+去甲基化药物联合治疗难治复发AML，继发AML缓解和生存也差于初发AML[28]。

此外，复发患者中，男性首次挽救化疗后缓解率低、缓解后OS期更短，这与我们既往报道初诊AML男性患者较女性患者缓解率低相一致[29]–[30]。这一结论还需更多病例研究的证实。

既往研究发现，难治复发AML患者年龄增加[26],[31]、根据遗传学特征进行危险度分层[6],[26]、早期复发[9],[25]、未接受移植[32]等均是不良预后因素，本研究也有同样发现。此外，本研究结果还显示，继发AML、复发时肿瘤负荷、性别也影响难治复发AML患者的预后。

本研究的局限性包括：①为单中心、回顾性研究；②本研究无联合细胞遗传学和基因组学进行危险度分层的数据，特别是在诱导化疗失败和复发时；③患者化疗方案多样，异质性较大；④收集本中心近20年的病例资料，年代跨度较大，治疗方案和支持治疗有差异；⑤因患者身体状态和经费等原因，并非所有患者都接受再诱导或移植等积极治疗，可能对缓解、复发和总结局有影响。

总之，本研究结果显示，诱导化疗失败和复发AML应用传统化疗疗效不佳，在无临床试验的条件下，强化疗方案再诱导、序贯移植可使患者获益。
